# AGIDB: a versatile database for genotype imputation and variant decoding across species

**DOI:** 10.1093/nar/gkad913

**Published:** 2023-10-27

**Authors:** Kaili Zhang, Jiete Liang, Yuhua Fu, Jinyu Chu, Liangliang Fu, Yongfei Wang, Wangjiao Li, You Zhou, Jinhua Li, Xiaoxiao Yin, Haiyan Wang, Xiaolei Liu, Chunyan Mou, Chonglong Wang, Heng Wang, Xinxing Dong, Dawei Yan, Mei Yu, Shuhong Zhao, Xinyun Li, Yunlong Ma

**Affiliations:** Key Laboratory of Agricultural Animal Genetics, Breeding, and Reproduction of the Ministry of Education & Key Laboratory of Swine Genetics and Breeding of the Ministry of Agriculture, Huazhong Agricultural University, Wuhan 430070, China; Key Laboratory of Agricultural Animal Genetics, Breeding, and Reproduction of the Ministry of Education & Key Laboratory of Swine Genetics and Breeding of the Ministry of Agriculture, Huazhong Agricultural University, Wuhan 430070, China; Key Laboratory of Agricultural Animal Genetics, Breeding, and Reproduction of the Ministry of Education & Key Laboratory of Swine Genetics and Breeding of the Ministry of Agriculture, Huazhong Agricultural University, Wuhan 430070, China; Key Laboratory of Agricultural Animal Genetics, Breeding, and Reproduction of the Ministry of Education & Key Laboratory of Swine Genetics and Breeding of the Ministry of Agriculture, Huazhong Agricultural University, Wuhan 430070, China; Key Laboratory of Agricultural Animal Genetics, Breeding, and Reproduction of the Ministry of Education & Key Laboratory of Swine Genetics and Breeding of the Ministry of Agriculture, Huazhong Agricultural University, Wuhan 430070, China; Hubei Hongshan Laboratory, Wuhan 430070, China; The Cooperative Innovation Center for Sustainable Pig Production, Huazhong Agricultural University, Wuhan 430070, China; Key Laboratory of Agricultural Animal Genetics, Breeding, and Reproduction of the Ministry of Education & Key Laboratory of Swine Genetics and Breeding of the Ministry of Agriculture, Huazhong Agricultural University, Wuhan 430070, China; Key Laboratory of Agricultural Animal Genetics, Breeding, and Reproduction of the Ministry of Education & Key Laboratory of Swine Genetics and Breeding of the Ministry of Agriculture, Huazhong Agricultural University, Wuhan 430070, China; Key Laboratory of Agricultural Animal Genetics, Breeding, and Reproduction of the Ministry of Education & Key Laboratory of Swine Genetics and Breeding of the Ministry of Agriculture, Huazhong Agricultural University, Wuhan 430070, China; Key Laboratory of Agricultural Animal Genetics, Breeding, and Reproduction of the Ministry of Education & Key Laboratory of Swine Genetics and Breeding of the Ministry of Agriculture, Huazhong Agricultural University, Wuhan 430070, China; Key Laboratory of Agricultural Animal Genetics, Breeding, and Reproduction of the Ministry of Education & Key Laboratory of Swine Genetics and Breeding of the Ministry of Agriculture, Huazhong Agricultural University, Wuhan 430070, China; Key Laboratory of Agricultural Animal Genetics, Breeding, and Reproduction of the Ministry of Education & Key Laboratory of Swine Genetics and Breeding of the Ministry of Agriculture, Huazhong Agricultural University, Wuhan 430070, China; College of Informatics, Huazhong Agricultural University, Wuhan 430070, China; Key Laboratory of Agricultural Animal Genetics, Breeding, and Reproduction of the Ministry of Education & Key Laboratory of Swine Genetics and Breeding of the Ministry of Agriculture, Huazhong Agricultural University, Wuhan 430070, China; Hubei Hongshan Laboratory, Wuhan 430070, China; College of Animal Science and Technology, Southwest University, Chongqing 402460, China; Key Laboratory of Pig Molecular Quantitative Genetics of Anhui Academy of Agricultural Sciences, Anhui Provincial Key Laboratory of Livestock and Poultry Product Safety Engineering, Institute of Animal Husbandry and Veterinary Medicine, Anhui Academy of Agricultural Sciences, Hefei 230031, China; College of Animal Science and Technology, Shandong Agricultural University, Taian 271018, China; Faculty of Animal Science and Technology, Yunnan Agricultural University, Kunming 650201, China; Faculty of Animal Science and Technology, Yunnan Agricultural University, Kunming 650201, China; Key Laboratory of Agricultural Animal Genetics, Breeding, and Reproduction of the Ministry of Education & Key Laboratory of Swine Genetics and Breeding of the Ministry of Agriculture, Huazhong Agricultural University, Wuhan 430070, China; Hubei Hongshan Laboratory, Wuhan 430070, China; Key Laboratory of Agricultural Animal Genetics, Breeding, and Reproduction of the Ministry of Education & Key Laboratory of Swine Genetics and Breeding of the Ministry of Agriculture, Huazhong Agricultural University, Wuhan 430070, China; Hubei Hongshan Laboratory, Wuhan 430070, China; Lingnan Modern Agricultural Science and Technology Guangdong Laboratory, Guangzhou 510642, China; Key Laboratory of Agricultural Animal Genetics, Breeding, and Reproduction of the Ministry of Education & Key Laboratory of Swine Genetics and Breeding of the Ministry of Agriculture, Huazhong Agricultural University, Wuhan 430070, China; Hubei Hongshan Laboratory, Wuhan 430070, China; Key Laboratory of Agricultural Animal Genetics, Breeding, and Reproduction of the Ministry of Education & Key Laboratory of Swine Genetics and Breeding of the Ministry of Agriculture, Huazhong Agricultural University, Wuhan 430070, China; Lingnan Modern Agricultural Science and Technology Guangdong Laboratory, Guangzhou 510642, China

## Abstract

The high cost of large-scale, high-coverage whole-genome sequencing has limited its application in genomics and genetics research. The common approach has been to impute whole-genome sequence variants obtained from a few individuals for a larger population of interest individually genotyped using SNP chip. An alternative involves low-coverage whole-genome sequencing (lcWGS) of all individuals in the larger population, followed by imputation to sequence resolution. To overcome limitations of processing lcWGS data and meeting specific genotype imputation requirements, we developed AGIDB (https://agidb.pro), a website comprising tools and database with an unprecedented sample size and comprehensive variant decoding for animals. AGIDB integrates whole-genome sequencing and chip data from 17 360 and 174 945 individuals, respectively, across 89 species to identify over one billion variants, totaling a massive 688.57 TB of processed data. AGIDB focuses on integrating multiple genotype imputation scenarios. It also provides user-friendly searching and data analysis modules that enable comprehensive annotation of genetic variants for specific populations. To meet a wide range of research requirements, AGIDB offers downloadable reference panels for each species in addition to its extensive dataset, variant decoding and utility tools. We hope that AGIDB will become a key foundational resource in genetics and breeding, providing robust support to researchers.

## Graphical Abstract

**Graphical Abstract:**
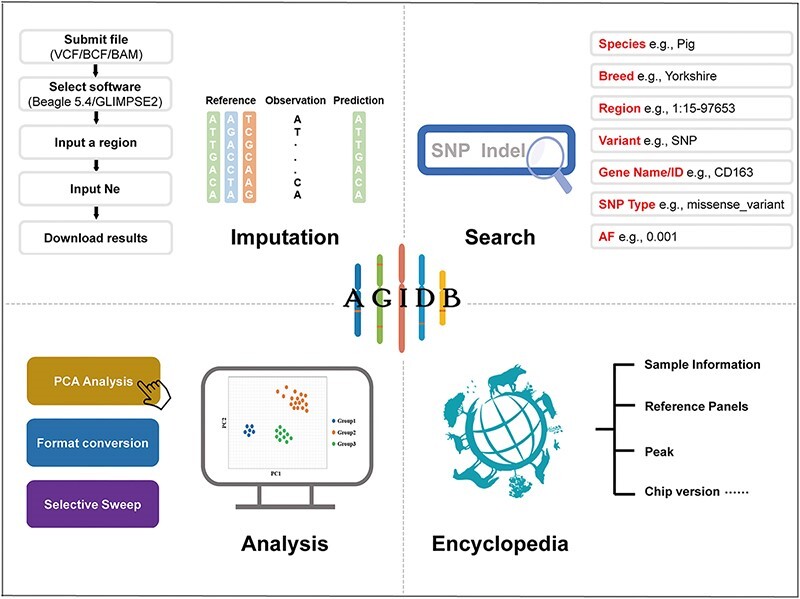

## Introduction

Advances in high-throughput sequencing technology have facilitated the rapid expansion of genetic decoding from humans and model animals to domestic and wild animals (1). This has led to the fine mapping of functional candidate genes, such as *DGAT1* (2) and *PROC* (3), as well as the development of genomic prediction, a marker-assisted selection technique (4). Both are facilitating significant advancements in animal breeding and precision medicine in humans. Although the cost of sequencing has decreased, research progress is hampered by sequencing expenses and the necessity of generating other omics data. The integration of low-coverage whole-genome sequencing and genotype imputation undoubtedly offers an optimal solution for achieving a balance between big data exploration and cost efficiency (5,6). Currently, lcWGS is a more cost-effective technology than low-density single nucleotide polymorphism (SNP) chips (5–7). Under ideal conditions, software such as Beagle 4.1 or GLIMPSE2 can easily achieve an accuracy rate of 95% or higher (5,8). Therefore, genotype imputation is an essential component of genomic data analysis and plays a crucial role in genetic research and biological breeding.

Since the initiation of the Human Genome Project, a series of publicly available datasets, including those of the International HapMap Project (9), the 1000 Genomes Project (10), the UK10K Project (11), the Haplotype Reference Consortium (HRC) (12), the Michigan Imputation Server (https://imputationserver.sph.umich.edu/) (13) and the Sanger Imputation Server (https://imputation.sanger.ac.uk/) (14), have been established to effectively harness genomic information.The fourth phase of the 1000 Genomes Project has been completed (15), and the sample size for human genetic research has already reached millions, with the number of markers exceeding tens of millions (13,14). Detailed functional annotation of the human genome, facilitated by high-performing researchers and sufficient research funding, has greatly advanced human genetics research. However, the construction, annotation, and mining of animal genome databases have a significant gap compared to human field. Therefore, for both domestic and wild animals, it is crucial to establish database platforms that can access each other's information across species and tools to make more effective use of limited data resources. Currently, the 1000 Bull Genomes Project (16) is a key bovine resource, while thousands of samples are available for many other domestic animals on the National Center for Biotechnology Information (NCBI) and European Bioinformatics Institute (EBI) platforms (17,18), and some animal-related databases, such as Animal-SNPAtlas (19), iDog (20), ISwine (21), IAnimal (22) and BGVD (23). Nevertheless, the sample sizes in the existing animal databases are often still too small, and deep data mining, such as for rare variants, conserved information between species, and population-specific variants, is still insufficient. The types of variants mainly consist of SNPs, with a few other types involving insertions and deletions (indels). As the benefits of genotype imputation in animals have become more apparent, database platforms such as Animal-ImputeDB (24) and the Aquaculture Molecular Breeding Platform (AMBP) (25) have been developed. However, while Animal-ImputeDB and AMBP contribute to genotype imputation, they are primarily focused on imputing from SNP chip data to sequencing data, with limited performance in scenarios requiring imputation from low-coverage to high-coverage whole-genome sequencing (WGS) data. Furthermore, these existing databases provide single, noncustomizable reference panels, inhibiting users from selecting and tailoring a reference panel subset that matches their specific datasets. In addition, the functional details of the existing databases, such as the effective population size and the reference panel, are not sufficiently considered, which can significantly affect the performance of genotype imputation if these parameters are used improperly or misused.

In this study, we developed an animal genotype imputation database and populated it with a sample size exceeding 192 000 individuals, encompassing 89 species and 1086 biological populations. The associated genotype imputation modules available on our website offer flexibility to meet practical application requirements, and the website enables searches for rare variants, conserved variants, deleterious variants, and population-specific variants, along with their annotation information in animal populations. Note that this is the first website that can be used to search for rare variants in animals. The website also includes expanded analysis modules, such as for genome characterization, principal component analysis (PCA), and selective sweep analysis modules. With a particular focus on lcWGS data, AGIDB offers an extensive reference panel, meticulously curated to cater to individual species and population structures. This feature enables users to tailor the reference panel subsets based on a specific breed or the population structure inherent to their dataset. Thus, AGIDB paves the way for a new era in cost-effective genetic research in animals, harnessing lcWGS data, pioneering imputation strategies, and exhaustive variant annotations. With its unique and flexible features, AGIDB enriches the landscape of existing databases, making it an invaluable resource for the genomics research community.

## Materials and methods

### Data sources

The WGS and chip data of 89 species, accompanied by genome annotation information, epigenomic data, and mined text information, such as species profiles and sources for each species, were systematically collected from global resources. Due to differences in the nature and scope of research among species, there are differences in the data collected for them, such as the number of subpopulations and the availability of Encyclopedia of DNA Elements (ENCODE) annotation information (26,27). Thus, we focused on collecting only representative samples to minimize data redundancy through information comparison and basic analysis. This approach is particularly useful for species with limited data, as it maximizes the amount of information gathered. Fundamentally, the data collection step involves obtaining raw WGS data and chip data from databases such as the SRA (https://www.ncbi.nlm.nih.gov/sra) (17), EBI (http://wwwdev.ebi.ac.uk/) (18), GSA (https://ngdc.cncb.ac.cn/gsa/) (28), Zenodo (https://zenodo.org/) (29), Dryad (https://datadryad.org/stash) (30), GEO (https://www.ncbi.nlm.nih.gov/geo/) (17) and Figshare (https://figshare.com/) (31) databases as well as from published literature. To expand the database, we generated 5086 whole-genome sequencing data and 8147 SNP chip data for pigs and chickens. Data collection was accompanied by an extensive curation process for associated BioProject data, including details on tissue, breed, and locality for each species. Text mining techniques were employed to extract details on the introduction and source of species and their subpopulations from Wikipedia, the Food and Agriculture Organization of the United Nations (FAO), and other sources. The reference genome and annotations of each species were obtained from the Ensembl database (https://asia.ensembl.org/index.html) (32), the MGI database (https://www.informatics.jax.org/) (33) and other databases. The final dataset, comprising approximately 688.57 TB of newly generated or collected data that underwent strict quality control, contained genome sequencing, SNP chip, ChiP-Seq and ATAC-Seq as well as genome annotation data for all species. Notably, the continuous generation of new data will ensure ongoing relevance of the AGIDB database.

### Data processing

- **Sequencing data processing**

The raw WGS reads were subjected to initial quality control assessment using FastQC v0.11.8 (34). The subsequent step involved data cleaning via Trimmomatic v0.39 to produce ‘clean’ reads (35). These reads were then aligned to relevant reference genomes using Burrows-Wheeler Aligner (BWA v0.7.17) mem (36). Following the alignment, the data were compiled into a binary alignment map (BAM) file. Any duplicates were identified and subsequently removed using the Picard tool of Genome Analysis Toolkit (GATK v4.1.9) (37). The process then proceeded with variant calling and refinement by HaplotypeCaller and VQSR, respectively. This led to the creation of an intermediate GVCF file for each individual. These GVCF files were further consolidated into a singular VCF file of raw variants using the GenotypeGVCFs tool in GATK (37). GATK was applied to raw SNPs for further filtering under specific parameters: ‘QUAL < 30.0, QD < 2.0, MQ < 40.0, FS > 60.0, SOR > 3.0, MQRankSum < –12.5, ReadPosRankSum < –8.0’. Biallelic SNPs and SNPs with a minimum sequencing depth of 5 were retained using VCFtools v0.1.16 (38). Subsequently, SNPs and indels that passed these filters were subjected to an additional round of quality control with PLINK v1.90 (39,40), which excluded SNPs and indels with a minor allele frequency (MAF) of <0.01 and a call rate of <0.9.

- **SNP chip data processing**

Based on the SNP chip data collected for each species, the positional information was initially adjusted according to the most recent version of the reference genome for that species. For example, the pig reference genome was standardized to Sus scrofa 11.1. Then, the SNP chip data were combined according to chip versions from various developers, and PLINK software was used for quality control with the parameters ‘–maf 0.01 –geno 0.1’. Finally, haplotype phasing was performed using Beagle 5.4 software with effective population size tailored for each species; for instance, an effective population size of 150 was deemed appropriate for pigs (8,41). In addition, map information for each species was collected from different SNP chip versions and developers, and standardized processing was based on chromosome and physical position. Venn diagrams were used to highlight the number of SNPs that overlap between different chips for different species.

- **Annotation resource processing**

The collected data, in addition to the genomic data, were mainly used as the contents of variant annotations in the ‘Search’ and ‘Analysis’ sections through text mining in this study. In the process of preparing annotation data, we utilized VCFtools v0.1.16 (38) to compute the allele frequency, performed LiftOver analysis to identify homologous sites, used SnpEff v5.0c software (42) for variant annotation, and employed SIFT and PolyPhen2 (43,44) software for predicting deleterious variants. To explore the specific characteristics of each breed (subpopulation) within a species, we defined breed-specific variant based on the following criteria: (a) the allele frequency of the variant in a particular breed was 1, while it was not 1 in all other breeds; (b) the allele frequency of the variant in a particular breed ranged from >0 to ≤1, while it was 0 in all other breeds and (c) the allele frequency of the variant in a specific breed was 0, while it differed from zero in all other breeds. Regarding the prediction of deleterious sites, we employed the default parameters and criteria without modification. All collected ChIP-Seq and ATAC-Seq datasets were first required to pass conversion and quality control with both fastp (V0.12.4) (34) and Chromap (V0.2.3) (45). MACS3 (V3.0.0a7) (46) was applied to identify peaks, employing specific parameters for ATAC-Seq data (‘-p 0.01 –nomodel –shift -75 –extsize 150 –keep-dup all -B –SPMR’) and ChIP-Seq data (‘-q 0.01 –nomodel –shift 0 –extsize $x –keep-dup all -B –SPMR’), with ‘$x’ calculated via SPP (V2.0.1) (47). We transformed the resulting bedGraph files to BigWig format using bedGraphToBigWig (V2.9) for downstream analysis (48).

### Functional module design

- **Versatile genotype imputation scenarios and reference panel construction**

In genotype imputation, Beagle demonstrated exceptional performance in haplotype phasing and chip data imputation, while GLIMPSE2 exhibited superior performance in lcWGS (5). Therefore, we integrated their respective unique advantages in constructing our database to cater to specific imputation requirements. According to our practical experience, we have developed four imputation modules that can effectively meet almost all potential application requirements (8). Among these, lcWGS imputation stands out as a module with promising growth potential. The integration of the ‘Genotype Imputation Reference Panel Intelligent Customization’ function and the ‘Format Conversion’ function within the ‘Tools’ and ‘Analysis’ sections enhances user convenience, providing a more streamlined user experience. The high-quality reference panels were assembled using Beagle 5.4 to phase high-quality SNPs for each of the species (49). These reference panels are available for local analysis and visualization in the ‘Download’ and ‘Genome Browser’ modules, respectively. Considering the massive and systematic data on pigs, we expanded and developed a platform with an expanded knowledge base (https://agidb.pro/AGIDB/Pig/Pig_home.php) for pigs, which provides users with a more refined selection of reference panels and incorporates more detailed popular science information.

- **Variant decoding and genomic features**

Based on the massive genomic dataset, the website functionality has been expanded to include variant decoding and genomic feature mining. These functional modules are implemented in the ‘Search’ and ‘Analysis’ sections. The decoding of variants focuses on breed-specific SNPs, SNPs conserved across species, deleterious variants, and rare variants. Tajima's D, Pi and F_ST_ are calculated simultaneously to detect selection signals (50), aiming to assess the changes in genomic features during the process of population adaptive evolution. By default, the window size for Tajima's *D* is 25 kb and for Pi/F_ST_, the window size and step size are 50 and 25 kb, respectively. This functional module comprises two components: database-driven and user-integrated data selective sweep analysis. We utilized DNA element information to annotate the variation sites in the database based on ChIP-Seq and ATAC-Seq data. LiftOver software was employed to establish connections between variant information for different species by annotating conserved information across species (51). This functionality facilitated interspecies coordinate conversions and enabled the identification of conserved SNP positions across multiple species, thereby offering valuable insights into shared genetic features and evolutionary relationships. To investigate the haplotype status of variants, we utilized LDBlockShow to visualize Linkage Disequilibrium (LD) blocks (52). Specifically, we used the following command: ‘LDBlockShow -InVCF ref.vcf.gz -Region [chrom]:[start]-[end] -OutPng -SeleVar 2 -OutPut test_LD_plot’.

- **A simplified version of the cross-species encyclopedia**

Popular science content has been incorporated into each section and module of the database to enhance the use of affinity analyses. We provide a brief introduction and representative pictures of each species, helping beginners in genome research learn about the origin and genetic relationships of species and their subpopulations. To elucidate the evolutionary relationships and genetic structure within and across the studied species, we utilized VCF2Dis v1.46 (37) and FigTree v1.4.3 (http://tree.bio.ed.ac.uk/software/figtree/) to construct phylogenetic trees. The resulting trees are available for download in the ‘Reference Panel’ module. In addition, we have developed a ‘Principal Component Analysis’ module that allows users to perform in-depth PCA using PLINK v1.90 (39,40). Here, our unique strategy seamlessly integrates user-contributed data with our carefully curated reference panels, enabling panoramic and nuanced PCA to capture the complexity of genetic variation. The above design facilitates the utilization of the database as a simplified version of the cross-species encyclopedia for users.

### Evaluation of imputation accuracy and the lcWGS imputation module

We chose the pig as a representative species to evaluate the performance of our lcWGS imputation module. We used SAMtools to downsample the BAM files of French Yorkshire pigs with an average coverage of 30× to depths of 0.1×, 0.3×, 0.5×, 0.7×, 1×, 2×, 3×, 4× and 5×. The effective population size was set to Ne = 150, while all other parameters were set to the default values of GLIMPSE2 and Beagle 5.4. Using these BAM files as input for GLIMPSE2, we calculated imputation accuracy in different MAF bins. Additionally, we conducted an imputation evaluation using actual 1× resequencing data obtained from 688 French Yorkshire pigs.

### Database implementation

AGIDB (https://agidb.pro) was developed utilizing the robust capabilities of the Nginx web server (v.1.22.1), complemented by PHP (v.7.4.33) and MySQL (v8.0.33) as its backend and database engine. The web interface has been optimized for superior performance across a wide range of web browsers, including but not limited to Chrome (recommended), Firefox, Internet Explorer, Opera, Microsoft Edge and Safari. The product offers unrestricted online access without mandatory registration.

## Results

### Data summary

**Figure 1.**
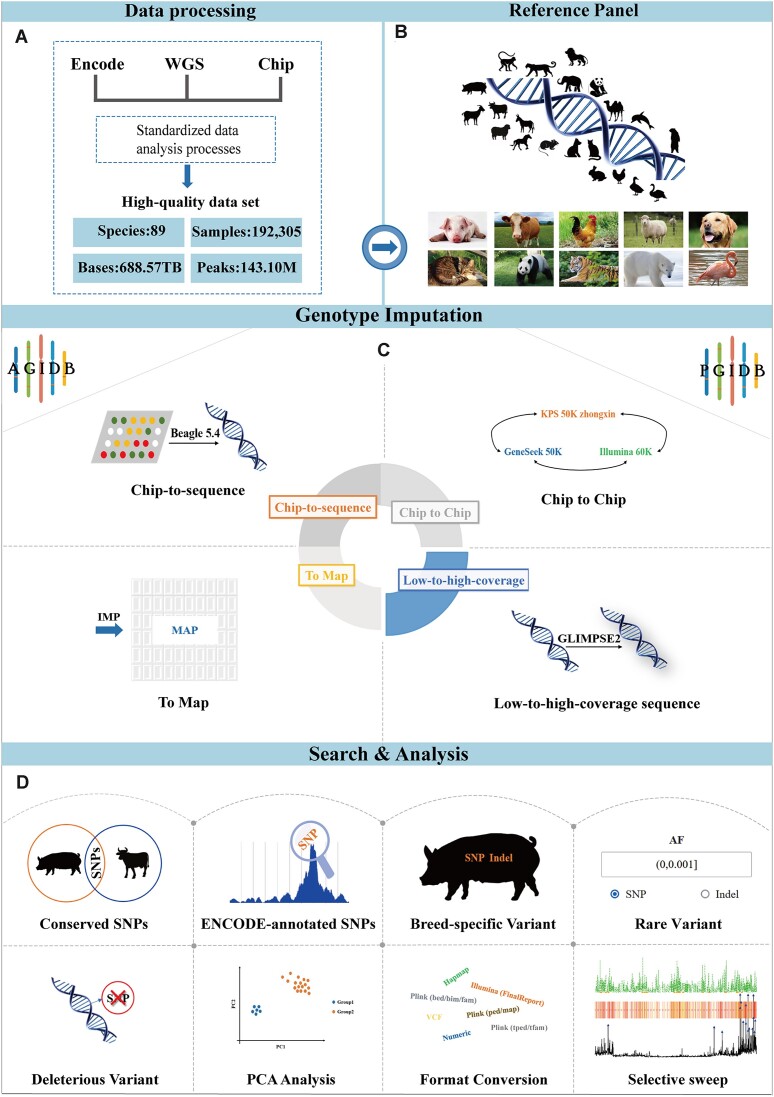
Overview of the AGIDB database. (**A**) Data information; (**B**) construction of reference panels; (**C**) genotype imputation modules; (**D**) search and analysis modules.

The present version of AGIDB is at the forefront of genotype imputation databases and unifies genomic data at an unprecedented scale (Table S1). We collected and compiled WGS data from 89 species, including a total of 17360 individuals from 1086 subpopulations (or breeds), to construct a haplotype reference panel for genotype imputation (Figure 1A, B). Compared with the existing genotype imputation databases, AGIDB includes almost 4 times as many species, has a more refined population division, and increases the sample size and data repository size by an order of magnitude (19,25). After quality control, a total of 688.57 TB of clean data from WGS was obtained, with the detection of 1245.8 million SNPs and 192.3 million indels. Additionally, we collected SNP chip data from 11 species, including 174 945 individuals, and constructed the SNP chip reference panel (Table 1). The phylogenetic tree, reference genome version, genome size, sample size, number of variants and detailed sample information for each species are displayed in the ‘Reference Panel’ module. Additionally, NCBI taxonomy numbers are provided to help users learn more about species. The database also includes ChIP-Seq and ATAC-Seq data from 10 species, along with multiple genome and genome annotation files for each species, to comprehensively annotate genomic variants.

**Table 1.** A partial summary of whole-genome sequencing and SNP chip data in *AGIDB*

Genus	Species	Population	Common name	Taxon ID	WGS	SNP chip
Sus	*Sus scrofa*	63	Pig	9823	6875	16085
	*Sus celebensis*	1	Celebes wild boar	273 789	10	
Bos	*Bos taurus*	29	Cattle	9913	979	137 381
	*Bos mutus*	1	Wild yak	72 004	108	
Bubalus	*Bubalus bubalis*	3	Water buffalo	89 462	30	618
Ovis	*Ovis aries*	148	Sheep	9940	875	8498
Capra	*Capra hircus*	104	Goat	9925	958	1944
Gallus	*Gallus gallus*	148	Chicken	9031	1501	1744
Meleagris	*Meleagris gallopavo*	1	Turkey	9103	29	
Equus	*Equus caballus*	56	Horse	9796	535	519
	*Equus asinus*	4	Donkey	9793	189	
Anas	*Anas platyrhynchos*	22	Mallard	8839	1161	
Anser	*Anser cygnoides*	7	Swan goose	8845	237	
Canis	*Canis lupus familiaris*	294	Dog	9615	2098	5919
	*Canis lupus*	9	Gray wolf	9612	15	73
Vulpes	*Vulpes rueppellii*	1	Rueppel's fox	354 189	25	
	*Vulpes vulpes*	1	Red fox	9627	38	
Felis	*Felis catus*	40	Cat	9685	310	1914
Panthera	*Panthera leo*	2	Lion	9689	39	
Oryctolagus	*Oryctolagus cuniculus*	13	Rabbit	9986	48	
Camelus	*Camelus dromedarius*	10	Arabian camel	9838	38	
Neovison	*Neovison vison*	9	American mink	452 646	17	
Ailuropoda	*Ailuropoda melanoleuca*	1	Giant panda	9646	55	
Ursus	*Ursus maritimus*	1	Polar bear	29 073	20	
	*Ursus thibetanus*	1	Asiatic black bear	9642	13	
Loxodonta	*Loxodonta africana*	1	African savanna elephant	9785	10	
Macaca	*Macaca mulatta*	6	Rhesus monkey	9544	696	
Theropithecus	*Theropithecus gelada*	1	Gelada	9565	18	
Mus	*Mus musculus*	14	House mouse	10 090	77	250
Camarhynchus	*Camarhynchus pallidus*	1	Woodpecker finch	48 878	10	
Certhidea	*Certhidea fusca*	1	Grey warbler finch	240 232	35	
	*Certhidea olivacea*	1	Green warbler finch	48 880	14	
Geospiza	*Geospiza conirostris*	1	Española cactus finch	48 882	47	
	*Geospiza difficilis*	1	Sharp-beaked ground finch	87 173	58	
Loxigilla	*Loxigilla noctis*	1	Lesser Antillean bullfinch	1 118 865	10	
Pinaroloxias	*Pinaroloxias inornata*	1	Cocos finch	93 070	20	
Platyspiza	*Platyspiza crassirostris*	1	Vegetarian finch	48 888	11	
**All** ^a^	**89 species**	**1086**			**17 360**	**174 945**

Note:

^a^The full list is provided in Table S1.

### Overview of AGIDB

AGIDB is a website integrated with a highly efficient, comprehensive and customized genomics database that focuses on the exploration of genetic resources, with its primary objective being the analysis of genetic diversity and genotype imputation serving as its core function (Figure 1). In the database, we comprehensively address the diverse requirements of users by incorporating a range of genotype imputation scenarios (Figure 1C). This is achieved through the ‘Imputation’ module, which allows users to select the most appropriate genotype imputation scenario according to their own research objects and requirements. Our goal is to provide users with accurate and personalized genotype imputation solutions.

To facilitate comprehension and thorough analysis of genomic information, we offer the ‘Search’ module (Figure 1D). This module presents a comprehensive range of query functions encompassing the encoding of annotated SNPs for each species, identification of conserved SNPs across species, analysis of breed-specific variants, detection of potentially deleterious variants in different populations, assessment of allele frequency distribution, exploration of haplotype blocks and utilization of genome browser tools. These functionalities aim to provide users with an extensive and comprehensive analysis of genetic variants. The ‘Analysis’ module integrates various functions, including PCA, genome file format conversion, and selective sweep analysis (Figure 1D). This provides users with robust support for analyzing complex genetic data in the AGIDB database. In addition, AGIDB also provides two auxiliary modules, ‘Download’ and ‘Help’. The ‘Download’ module facilitates effortless download of reference panels for each species by chromosome, while the ‘Help’ module offers comprehensive instructions and tutorials to aid users in understanding and mastering AGIDB. On the basis of AGIDB, we also developed PGIDB as an extension of AGIDB, which is specially designed for in-depth exploration of the pig genome and breeding applications. It will play a crucial role in facilitating genotype imputation and variant annotation services specifically tailored for pig breeding, offering researchers the choice of 30×, 10× and chip-based panels. Overall, AGIDB is a comprehensive platform that deeply integrates various genomics tools and data, providing users with an efficient and professional one-stop genome annotation service while also offering robust tool support for advancing genomics research and applications.

### Genotype imputation modules

**Figure 2.**
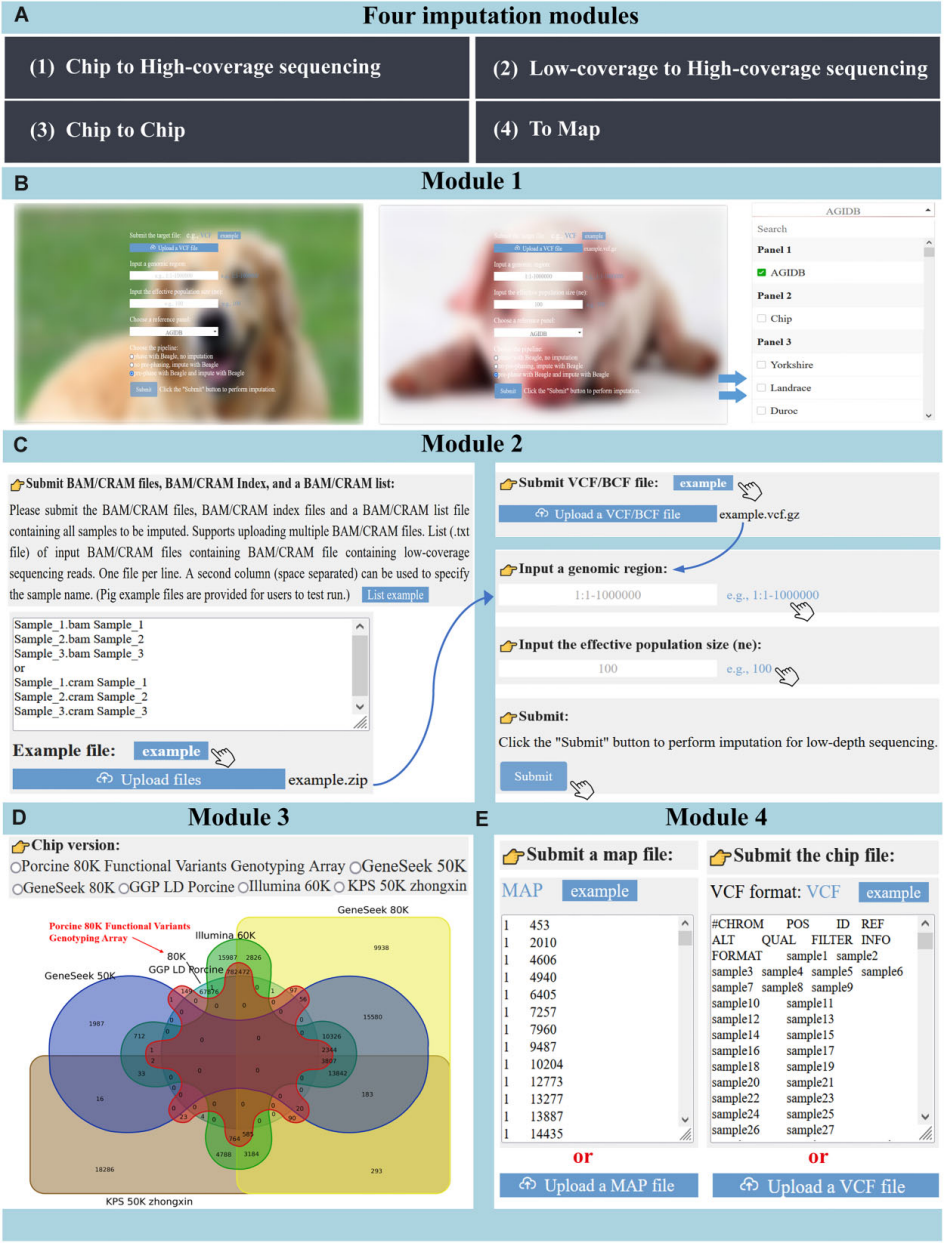
Schematic diagram of genotype imputation modules. (**A**) Scenarios of imputation in AGIDB; (**B**) the interface and flowchart of imputation from chip to high-coverage sequencing; (**C**) the interface and flowchart of imputation from low-coverage to high-coverage sequencing; (**D**) the interface and flowchart of imputation from chip to chip; (**E**) the interface and flowchart of imputation to a specific map.

In genomics research, genotype imputation not only enhances SNP density and improves the efficacy of genome-wide association studies but also facilitates the identification of causative mutations and reduces the cost of genomic prediction (53,54). AGIDB includes four meticulously developed novel genotype imputation modules for this purpose. Because users have different requirements for genotype imputation (8), the four genotype imputation modules (Figure 2A) include chip-to-sequencing (Figure 2B), low-coverage to high-coverage sequencing (Figure 2C), conversion between different chip versions (Figure 2D), and imputing to a user-specified map (Figure 2E). Based on these genotype imputation modules, users can select the most appropriate imputation module according to their specific requirements. Furthermore, users have the option to utilize a comprehensive reference panel containing all individuals or select a specific reference panel based on their requirements. This not only enhances the accuracy of genotype imputation but also optimizes computational resource allocation. Using the pig as an example, available reference panels include those encompassing all individuals, chip-based reference panels, and breed-specific reference panels. If users have their own computing platform, they can download a reference panel for genotype imputation. In this way, AGIDB will facilitate subsequent genomics research.

**Figure 3.**
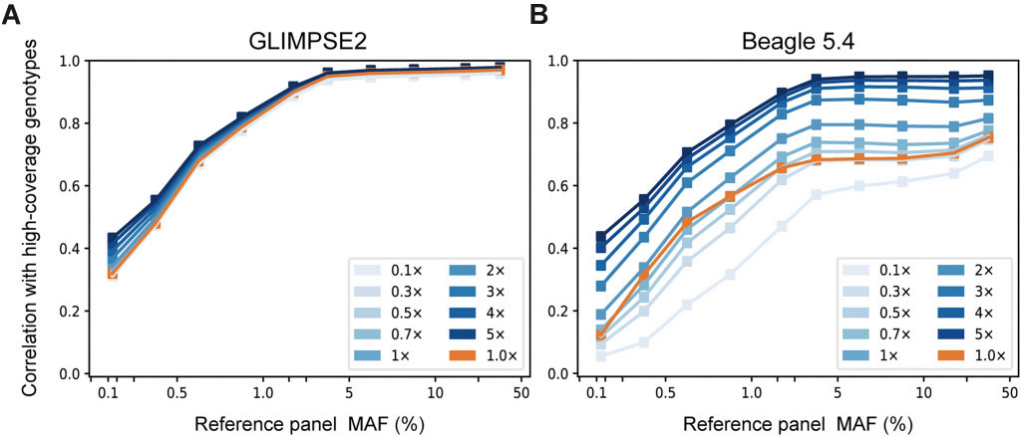
The performance of genotype imputation from low-coverage to high-coverage sequencing. Using sequencing data derived from nine different downsampling scenarios (0.1×, 0.3×, 0.5×, 0.7×, 1×, 2×, 3×, 4× and 5×) and real 1× scenario, we compared the imputation accuracies of Beagle and GLIMPSE2. Here, we used Beagle 4.1 for phasing and subsequently performed genotype imputation using Beagle 5.4.

For the module supporting imputation from low-coverage sequencing to high-coverage sequencing, we employed GLIMPSE2, a tool capable of directly processing BAM files, which allows users to bypass the genotype calling step and perform direct genotype imputation. The optimization enhances the efficiency of imputation and facilitates time savings for users. Based on simulated low-coverage sequencing data and real low-coverage sequencing data, our results show that GLIMPSE2 has a high imputation accuracy for common variants (MAF > 0.05) at 1× or even lower sequencing coverage (Figure 3A). Therefore, the integration of our database and GLIMPSE2 has yielded promising results in terms of genotype imputation accuracy. This demonstrates the efficacy of utilizing genotype imputation to facilitate the use of low-coverage sequencing data in genetics and breeding, leveraging dependable database resources (5).

For the other imputation modules, we have selected Beagle 5.4 as our preferred method. To further enhance the accuracy of genotype imputation, we offer customized settings for effective population size, which has been demonstrated to play a crucial role in determining imputation accuracy for a given species (55). In the genotype imputation modules, users can easily upload a VCF file or select a sample file, choose from default or customized reference panels, specify the genome region of interest and input an effective population size for genotype imputation. Although Beagle exhibits lower accuracy than GLIMPSE2 for low-coverage imputation, it still demonstrates remarkably high imputation accuracy for cases with sequencing coverage above 4× (Figure 3B). Furthermore, previous studies have consistently highlighted Beagle's notable advantages in terms of imputation speed and its proven reliability in SNP chip imputation (8,25,55).

Notably, the flexibility of AGIDB genotype imputation is reflected in the fact that users can meet personalized imputation needs by calling different modules to combine functions. For example, if there are no overlapping SNPs between different versions of SNP chips, users can still use the ‘chip to high-coverage sequencing’ module and the ‘to map’ module to impute a target SNP chip version, which is not available in any other existing genotype imputation database. After genotype imputation, users can access and download the resulting data for further analysis. This design not only enables our genotype imputation module to support a variety of imputation scenarios but also enhances its usability and efficiency, thereby providing robust support for users’ genomics research. In addition, AGIDB ensures the simultaneous operation of multiple users and the security of user data by maintaining exclusive accessibility to uploaded data for imputation and analysis by the uploader. Finally, the uploaded data is automatically deleted within 7 days after the completion of imputation task to optimize data storage space.

### Search modules

#### Breed-specific variant search module

**Figure 4.**
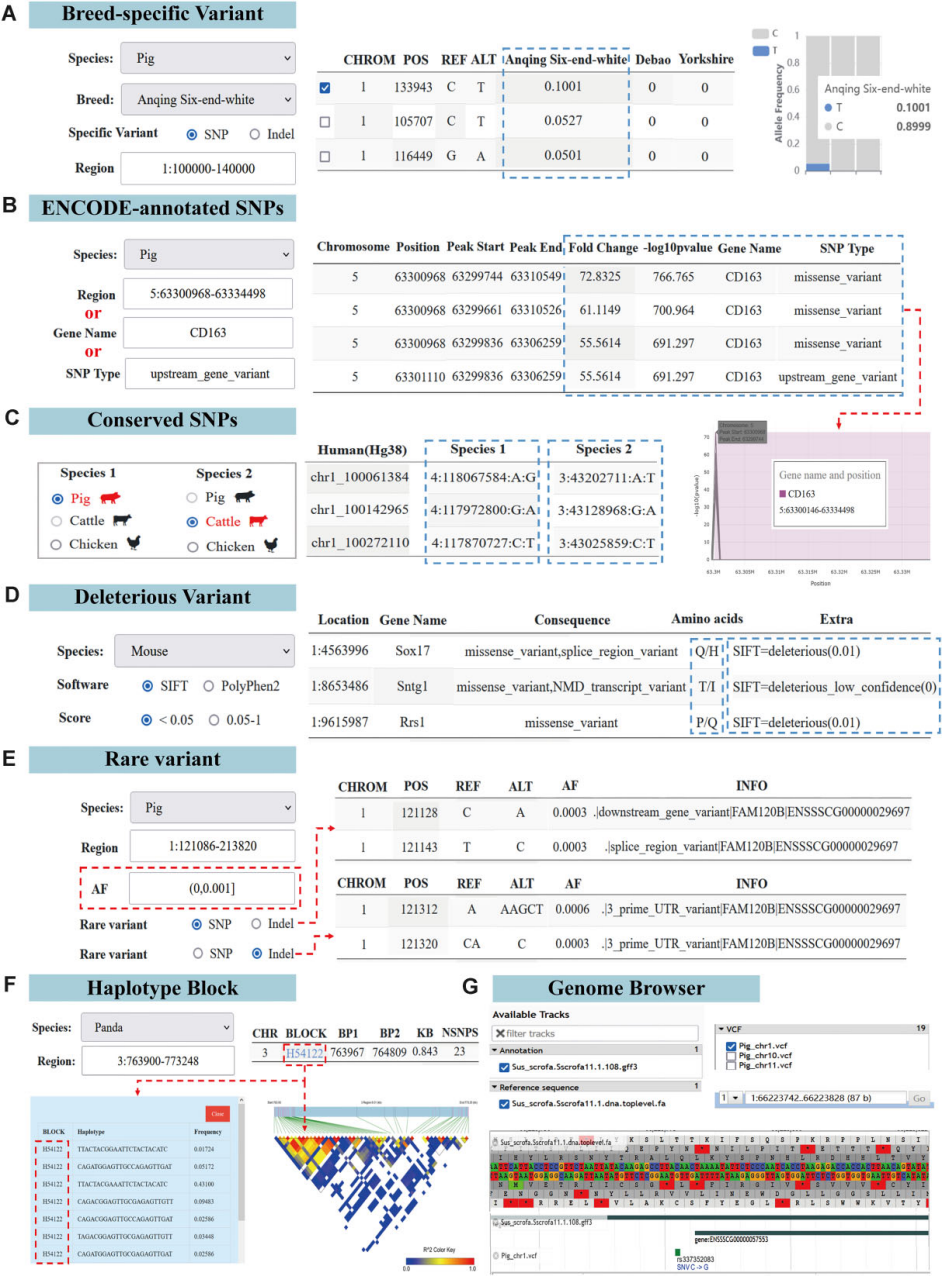
Variant decoding and search modules. (**A**) Searching for breed-specific variant; (**B**) searching for ENCODE-annotated SNPs; (**C**) searching for conserved SNPs; (**D**) searching for deleterious variant; (**E**) searching for rare variant; (**F**) haplotype block detection and visualization; (**G**) variant visualization.

In the ‘Breed-specific Variant’ module, users can select a species and its subpopulation (breed) and input a specific region of interest in the genome, and then the query system will return the corresponding breed-specific SNPs within that region (Figure 4A). This module provides researchers with an efficient way to quickly identify and locate specific SNPs that may affect breed characteristics in many breeds, helping them understand how these SNPs drive genetic diversity and adaptation among the breeds. Here, users can query allele frequencies for all SNPs within a given region and their distribution across various breeds. For more targeted queries, users can customize subpopulations to obtain corresponding results specific to a particular breed. Furthermore, the module offers a visualization of allele frequency distribution to provide users with a more intuitive understanding of how a specific SNP is distributed across breeds (Figure 4A). The development and implementation of this feature can contribute to our comprehension and preservation of the genetic diversity within species.

#### ENCODE-annotated SNPs search module

In the ‘ENCODE-annotated SNPs’ module, users can enter specific genomic regions, such as “5:63300968–63334498”, to search for SNPs and obtain annotation details. The example search results revealed 2 SNPs that overlapped with the potential regulatory element. One of these SNPs, located at locus 63300968 on chromosome 5, exhibited a markedly high fold change value of 72.8325 and a -log_10_*P* value of 766.765 (Figure 4B). When considering the variant annotation information, this implies that the SNP might not only be a missense variant in the *CD163* gene but also a distant regulator of other genes, indicating its potential importance. A previous study found that *CD163* is closely associated with porcine reproductive and respiratory syndrome infection (56). However, the complex pathogenesis of porcine reproductive and respiratory syndrome is still not fully understood (57). Therefore, the design of this module in our database could offer references for further research on porcine reproductive and respiratory syndrome and provide insights into the genetic mechanisms of other complex traits. Furthermore, users can quickly and accurately obtain the same results by entering a specific gene ID, such as ‘CD163’, or the SNP type ‘upstream_gene_variant’; this feature significantly enhances the utility and user experience of our database (Figure 4B).

### Conserved SNPs search module

The ‘Conserved SNPs’ module presents conserved SNPs among 9 species that can be mapped to the human genome. The user simply selects two species, such as pig (species 1) and cattle (species 2), and then clicks ‘Search’, after which the system will list the human (Hg38) coordinates in the table, along with the corresponding SNP coordinates and mutation information for the two selected species. For example, the SNP at position 100 061 384 bp on human chromosome 1 corresponds to position 118 067 584 bp on pig chromosome 4, where a mutation from A to G occurs. Similarly, in cattle, this SNP corresponds to position 43 202 711 bp on chromosome 3, with a mutation from A to G (Figure 4C). These organized data allow researchers to clearly observe the specific location and variation of the SNP across different species, shedding light on its possible functional conservation.

### Deleterious variant search module

In the ‘Deleterious Variant’ module, PolyPhen2 and SIFT are used to predict deleterious variants across the whole genome. After selecting species and breeds, the user chooses the software for predicting deleterious variants and sets the corresponding threshold for defining deleterious variants. Then, once the user clicks ‘Search’, the system will return a table that includes information such as mutation location, alleles, affected genes, and predicted scores to comprehensively present potentially deleterious variant information. For example, using SIFT software with a threshold of 0.05 revealed a missense mutation site at chr1:9615987 within the coding region of the *Rrs1* gene, resulting in an amino acid substitution from proline to glutamine (Figure 4D). According to the prediction by SIFT (predicted score: 0.01), this variant demonstrates the highest level of deleteriousness, and previous research has established that the *Rrs1* gene serves as a potential molecular biomarker for mouse model of Huntington disease (58).

### Rare variant search module

In the ‘Rare Variant’ module, rare variants are defined as SNPs and indels with allele frequencies less than or equal to 0.005. Users can select the species of interest to search for rare variations within a specified genomic region. By default, the system displays all rare variants with allele frequencies between 0 and 0.005, along with their annotation information. Users can also customize variant types and allele frequency thresholds (e.g. (0,0.001]) to search for more targeted rare SNPs and indels (Figure 4E). The module provides a convenient platform for researchers to understand rare variants in specific genomic regions of particular species, thereby contributing to in-depth research and understanding of the possible functional effects of these variants.

### Haplotype block and visualization module

In the ‘Haplotype block’ module, users can query haplotype blocks and visualize LD heatmaps. When species and genomic regions are selected, the system will display corresponding haplotype block results. These results are presented in table form on the page, and by clicking on the haplotype block ID in the table, users can view detailed information about the haplotype and its frequency. For example, when the panda genome region “3:763900–773248” is queried, a total of 5 haplotype blocks are returned. Among them, the haplotype block ‘H54122’ contains 23 SNPs and 10 haplotype distributions. When the ‘LD Plot’ button is clicked, the system generates an LD heatmap of the region. These 23 SNPs are closely linked to form a region with a high LD value, that is, a large haplotype block (Figure 4F). This module allows for an easy and accurate understanding of genetic linkage information in specific genomic regions, which is significant for exploring the genetic basis of complex traits.

### Variant visualization module

In the ‘Genome Browser’ module, users can click on a specific species’ image to select it. Then, in the left section of the Genome Browser (JBrowse), users can autonomously choose various track information, such as reference genomes, reference genome annotations, reference panels divided by chromosome, and indels. This provides a personalized view of the genomic data. In the right-hand section of JBrowse, users can see detailed visualizations of specific genomic regions (Figure 4G). This visualization method is an intuitive and easy method for locating and identifying genes or SNPs. Users simply left-click on a specific gene or SNP to obtain comprehensive information about it, including its basic properties, detailed descriptions, genotype distribution, and genotype information in a specific sample.

### Analysis modules

#### Principal component analysis module

**Figure 5.**
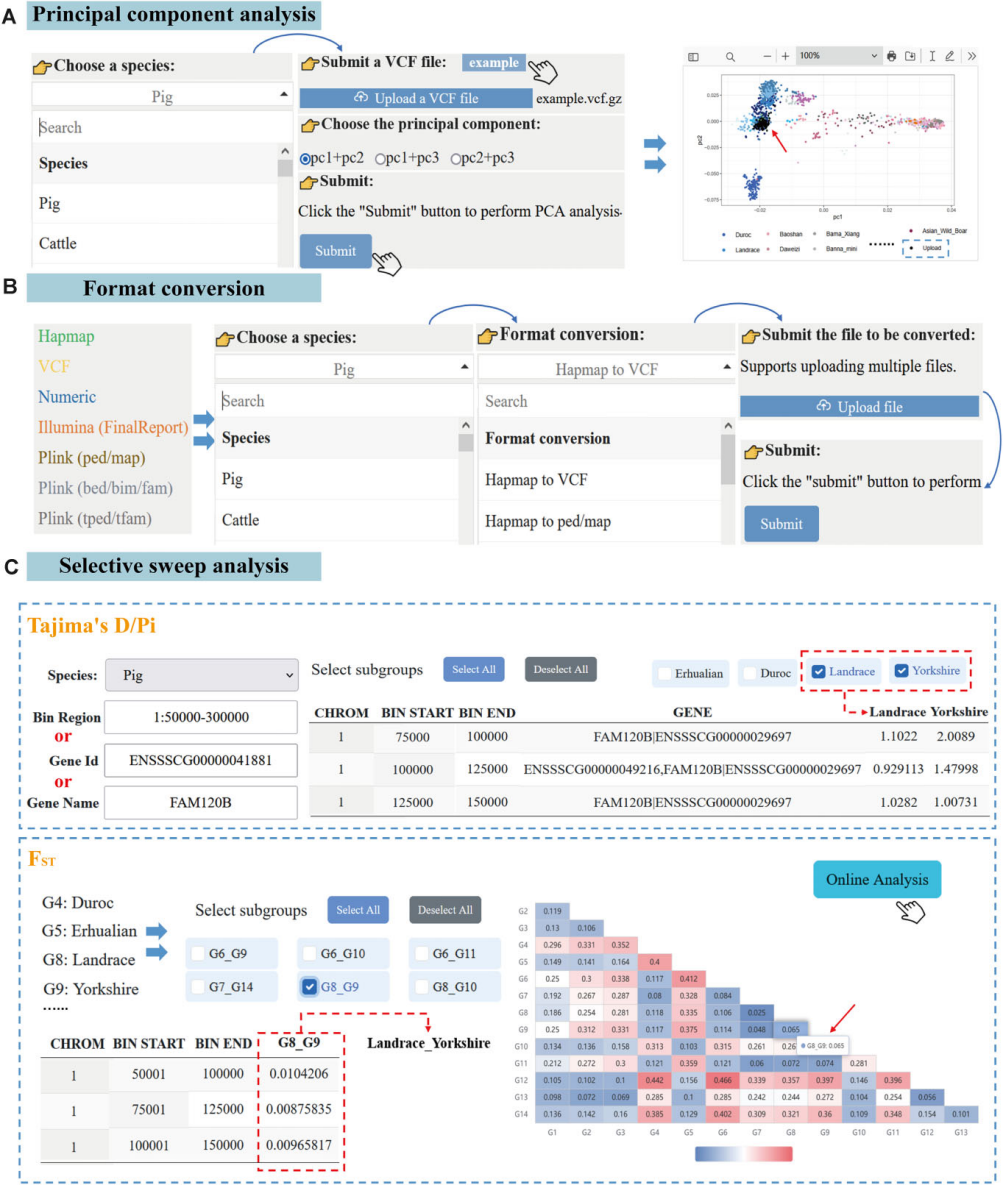
The main functions and usage of the analysis section. (**A**) Principal component analysis; (**B**) genotype data format conversion; (**C**) selective sweep analysis.

In the ‘Principal Component Analysis’ module, we provide a series of preset sample files for each species to assist users in conducting PCA. Users have the option to either utilize these preset sample files or upload custom VCF files. Additionally, users can select the desired principal component based on their specific requirements. Once the Submit button is clicked, the system will promptly perform PCA and generate a clear PCA chart. The sample files or samples from user-uploaded files will be clearly indicated by black dots in this chart for easy identification by users. For pigs specifically, we choose a preset example file for PCA and generate a PCA plot displaying the 1st and 2nd principal components (PC1 and PC2, respectively) (Figure 5A). The generated PCA diagram is available for direct download. This functional module can help users visualize the location of their target population in a broader context, incorporating more information about other populations into the framework to maximize data utilization.

#### Genotype data format conversion module

In the ‘Format Conversion’ module, we provide downloadable sample files in seven major genomic data formats, including Hapmap, VCF, Numeric, Illumina (FinalReport), Plink (ped/map), Plink (bed/bim/fam), and Plink (tped/tfam) (Figure 5B). Considering that different researchers may use different data formats for specific research practices, we have set up 12 possible conversion options to cover common conversion requirements between the above seven formats. When the user selects the appropriate conversion option and submits the request, the converted file is automatically displayed on the current page of their browser for immediate download and subsequent analysis. The design of this module enables users to easily and quickly perform conversions between various genomic data formats while improving efficiency and convenience in genetic data processing and application.

#### Selective sweep analysis module

In AGIDB, the ‘Selective Sweep Analysis’ module provides users with detailed calculations and interpretations of Tajima's D, Pi and F_ST_, which are three important genetic statistical indicators used for detecting selective sweeps (Figure 5C). Users can obtain Tajima's D/Pi values for each subpopulation of the selected species by specifying the species name, bin region, gene ID, or gene name. By default, the system displays Tajima's D/Pi values for all subpopulations of the species. However, users can also select specific subpopulations to obtain their corresponding Tajima's D/Pi values based on research needs.

Additionally, the F_ST_ section of the ‘Selective Sweep Analysis’ module presents *F*_ST_ values between subpopulations with a sample size greater than 20, enabling users to select specific pairs of subpopulations and obtain their respective *F*_ST_ values. Furthermore, to enhance data interpretation for users, we have incorporated an *F*_ST_ heatmap that visually represents the genetic distance between pairs of subpopulations. For example, the *F*_ST_ value between subpopulations G5 (Erhualian pig) and G9 (Yorkshire pig) is 0.375, while that between subpopulations G8 (Landrace pig) and G9 (Yorkshire pig) is 0.065, indicating high genetic similarity between Landrace and Yorkshire pigs. Overall, this module provides researchers with an efficient and practical set of tools for understanding genetic differences and selective sweeps within and across species, thereby enhancing our understanding of genetic relationships among species as well as natural selection processes.

### A simplified version of the cross-species encyclopedia

In the ‘Reference Panel’ module of AGIDB and the ‘Library’ module of the extended database PGIDB, we provide a brief introduction to each species. Users can access comprehensive information on each species and its subpopulations, encompassing Latin nomenclature, taxonomic classification, genome size, chromosome number, and well-known genes. Using pigs as an example, we present a comprehensive taxonomy that encompasses 5 genera and 10 species, accompanied by detailed descriptions of 63 breeds along with photographs and concise introductions. We also incorporate the latest relevant literature and databases to provide beginners with a comprehensive understanding of the study object and its closely related species.

### Tools, download and others section

The ‘Tools, download and others’ section integrates various genomic analysis tools and offers comprehensive data downloads, including sample information sheets, reference panels, epigenomics peak data, and chip maps. To foster a dynamic and reciprocal relationship with users, AGIDB incorporates a data upload module, eliminating the need for user registration or login. This innovative feature enables users to simply submit data links, which are systematically archived with timestamps on our backend. This not only ensures prompt and seamless updates of our database but also illustrates our commitment to nurturing a synergistic platform for continuous data enrichment and evolution. Furthermore, comprehensive usage documentation is provided to enhance user proficiency in making use of our resources and services.

## Summary and future directions

The present study provides an overview of the development of AGIDB, a comprehensive multispecies genotype imputation and genetic information integration database. AGIDB demonstrates outstanding advantages in comparison to other previously published databases with similar functionalities. First, AGIDB is a pioneering database that offers comprehensive expansion of genotype imputation modules. In addition to conventional chip-to-sequencing imputation, it also incorporates low-coverage sequencing imputation and enables conversion between various chip versions. Moreover, it facilitates imputation to a specified map and other modules, thereby achieving complete coverage of genotype imputation functionality. Furthermore, AGIDB includes a novel Custom Reference Panel module, enabling users to create and utilize a dedicated reference panel tailored to their specific needs, thereby facilitating a broader range of genotype imputation applications. Finally, the ‘Search’ module offers a novel way to integrate and present diverse genomic information, including breed-specific SNPs, deleterious variants, conserved SNPs, haplotype blocks, and more detailed variant annotations. This facilitates the establishment of interspecies and interbreed connections to promote a comprehensive understanding and in-depth exploration of genetic variation (Table 2).

**Table 2.** Functional innovations of AGIDB compared with other databases

Functions	AGIDB	Animal-ImputeDB (24)	Plant-ImputeDB (61)	Animal-SNPAtlas (19)	AMBP^a^ (25)	SWIM (62)	PHARP (63)
**Reference panel**							
Range: multispecies	√	√	√	√	√		
Precision: breed/line	√						
**Search/Analysis**							
Conserved SNPs	√						
Deleterious Variant	√						
Rare Variant	√						
Haplotype Block	√		√	√			
Genome Browser	√			√			
**Imputation**							
Chip-to-sequence	√	√	√	√	√	√	√
Low-coverage sequence	√				√		
Chip to Chip	√						
To Map	√						
ImpRefIC^b^	√						
**Total numbers**	**12**	**2**	**3**	**4**	**3**	**1**	**1**

Note:

^a^AMBP represents the Aquaculture Molecular Breeding Platform;

^b^ImpRefIC represents Genotype Imputation Reference Panel Intelligent Customization.

In the future, we intend to continue maintaining and developing AGIDB. With the rapid development of sequencing technology, we will actively integrate novel sequencing data, including proactively generating data to enhance the species and population coverage of our reference panels. Simultaneously, the emerging field of pan-genomics offers new avenues and challenges for genotype imputation and variant decoding (59,60). To meet the current demands of genetic research, it is no longer sufficient to focus solely on genotype imputation of SNPs. Therefore, we aim to expand our imputation capabilities to include other types of genetic variants, such as indels, providing more comprehensive support for genetic analysis. Additionally, we will continue to develop a comprehensive set of application modules to provide one-stop services such as species knowledgebase construction, variant decoding, genotype imputation and post-imputation data application, making AGIDB a truly all-encompassing animal genetic information service platform.

## Supplementary Material

gkad913_Supplemental_File

## Data Availability

AGIDB is freely available to the public at https://agidb.pro, and it can also be accessed through the mirror website http://animal_breeding_lab.hzau.edu.cn/AGIDB.
