# Supplementary material for: AGIDB: a versatile database for genotype imputation and variant decoding across species
Source: Nucleic Acids Res. 2023 Oct 27;52(D1):D835–49. doi: 10.1093/nar/gkad913 (PMC10767904; doi:10.1093/nar/gkad913)
Supplement: gkad913_Supplemental_File [file gkad913_supplemental_file.pdf]

Table S1. Summary of whole genome sequencing and SNP chip data in *AGIDB*.

Genus	Species[1]	Populations/Breeds	Common Name	Taxon Id	WGS	Depth	SNPs	Indels	Bases	SNP chip
Sus	*Sus scrofa*	63	Pig	9823	6,875[2]	4.25	20,954,035	2,340,078	72.99TB	16,085
	Sus barbatus	1	Bearded pig	41807	4	11.25	9,009,084	1,847,548	112.52GB	
	Sus cebifrons	1	Visayan warty pig	315377	10	14.12	4,862,142	1,107,681	352.91GB	
	Sus celebensis	1	Celebes wild boar	273789	3	12.44	4,762,753	984,072	93.3GB	
	Sus verrucosus	1	Java warty pig	159856	4	20.81	1,112,743	383,546	208.14GB	
Babyrousa	*Babyrousa babyrussa*	1	Babirusa	41806	1	11.12	352,689	151,057	27.79GB	
Phacochoerus	*Phacochoerus africanus*	1	Common warthog	41426	2	12.36	1,877,242	626,332	61.81GB	
Porcula	*Porcula salvania*	1	Pygmy hog	476284	7	16.75	18,886,770	3,601,154	293.14GB	
Potamochoerus	*Potamochoerus larvatus*	1	Bushpig	273792	1	11.3	1,342,030	446,833	28.25GB	
	Potamochoerus porcus	1	Red river hog	273791	1	10.71	2,464,707	744,970	26.77GB	
Bos	*Bos taurus*	29	Cattle	9913	979	17.03	13,018,725	1,762,927	49.06TB	137,381
	Bos indicus	1	Zebu cattle	9915	4	12.93	21,357,497	2,314,410	134.24GB	
	Bos grunniens	35	Domestic yak	30521	108	9.17	13,080,255	2,181,769	2.84TB	
	Bos mutus	1	Wild yak	72004	6	35.56	13,405,765	2,028,666	597.42GB	
Bubalus	*Bubalus bubalis*	3	Water buffalo	89462	30	18.97	22,885,523	2,295,159	1.12TB	618
Ovis	*Ovis aries*	148	Sheep	9940	875	13.35	26,524,394	3,548,656	35.16TB	8,498
	Ovis vignei	1	Urial	59896	1	16.92	9,861,976	1,125,828	59.15GB	
Capra	*Capra hircus*	104	Goat	9925	958	15.33	19,043,788	2,228,053	49.25TB	1,944
	Capra aegagrus	1	Wild goat	9923	3	11.87	11,835,009	1,325,445	101.33GB	
Gallus	*Gallus gallus*	148	Chicken	9031	1,501	14.92	14,754,681	1,692,127	19.53TB	1,744
	Gallus lafayetii	1	Ceylon junglefowl	9032	2	13.7	2,899,737	327,017	30.96GB	

Meleagris	*Meleagris gallopavo*	1	Turkey	9103	29	5.15	4,285,866	516,862	122.50GB	
Columba	*Columba guinea*	1	Speckled pigeon	135627	1	33.87	3,092,875	359,043	40.64GB	
	Columba larvata	1	Eastern lemon Dove	1804667	1	26.6	10,653,584	1,262,599	31.92GB	
	Columba leucomela	1	White-headed Pigeon	471126	1	21.22	6,640,190	711,777	25.46GB	
	Columba livia	1	Rock pigeon	8932	2	14.84	5,698,851	835,209	35.61GB	
	Columba palumbus	1	Common wood Pigeon	8934	1	34.88	4,058,500	455,271	41.86GB	
	Columba rupestris	1	Hill pigeon	126356	1	164.38	5,184,281	716,339	197.26GB	
Equus	*Equus caballus*	56	Horse	9796	535	18.23	11,621,030	1,188,974	28.39TB	519
	Equus asinus	4	Donkey	9793	189	3.62	2,391,751	375,005	1.94TB	
Anas	*Anas platyrhynchos*	22	Mallard	8839	1,161	6.86	14,033,546	2,433,610	10.89TB	
Anser	*Anser cygnoides*	7	Swan goose	8845	237	9.64	4,027,944	517,032	3.02TB	
Canis	*Canis lupus familiaris*	294	Dog	9615	2,098	20.66	8,763,103	2,490,533	107.54TB	5,919
	Canis lupus	9	Gray wolf	9612	15	23.84	377,299	93,587	796.50GB	73
	Canis lupus baileyi	1	Mexican gray wolf	143281	1	11.43	68,528	16,118	236.63GB	
	Canis lupus lupus	2	Eurasian wolf	443256	4	19.91	8,968,166	2,153,062	235.37GB	
Vulpes	*Vulpes pallida*	1	Pale fox	1224817	5	1.9	4,127,961	1,109,465	22.75GB	
	Vulpes rueppellii	1	Rueppel's fox	354189	25	0.95	10,756,466	3,403,194	57.03GB	
	Vulpes vulpes	1	Red fox	9627	38	0.71	14,834,890	4,912,198	81.61GB	
	Vulpes zerda	1	Fennec fox	68732	5	4.29	2,214,366	716,704	51.53GB	
Felis	*Felis catus*	40	Cat	9685	310	26.86	21,280,882	4,515,762	22.26TB	1,914
Herpailurus	*Herpailurus yaguarondi*	1	Jaguarundi	1608482	1	28	756,448	189,311	70.54GB	

Panthera	*Panthera leo*	2	Lion	9689	39	8.94	2,816,827	1,320,538	910.81GB	
	Panthera pardus	1	Leopard	9691	4	24.27	5,226,516	1,022,042	240.28GB	
	Panthera tigris	1	Tiger	9694	6	36.14	447,072	239,086	657.51GB	
Oryctolagus	*Oryctolagus cuniculus*	13	Rabbit	9986	48	11.01	37,007,357	6,191,917	2.16TB	
Lepus	*Lepus americanus*	1	Snowshoe hare	48086	1	24.55	45,579,072	6,307,162	78.63GB	
Vicugna	*Vicugna pacos*	1	Alpaca	30538	8	31.21	2,787,853	18,500,239	524.35GB	
Camelus	*Camelus dromedarius*	10	Arabian camel	9838	38	15.27	3,832,243	807,965	1.39TB	
Neovison	*Neovison vison*	9	American mink	452646	17	22	9,103,205	2,441,640	1.01TB	
Ailuropoda	*Ailuropoda melanoleuca*	1	Giant panda	9646	55	6.71	7,366,555	1,595,775	1.26TB	
Ursus	*Ursus maritimus*	1	Polar bear	29073	20	3.6	1,929,991	680,647	165.71GB	
	Ursus thibetanus	1	Asiatic black bear	9642	13	3.54	3,454,636	624,920	141.69GB	
	Ursus americanus	1	American black bear	9643	1	72.97	1,919,773	528,872	232.86GB	
Loxodonta	*Loxodonta africana*	1	African savanna elephant	9785	10	20.05	7,499,561	1,248,557	810.38GB	
Dugong	*Dugong dugon*	1	Dugong	29137	1	17.58	2,151,038	259,758	110.83GB	
Macaca	*Macaca mulatta*	6	Rhesus monkey	9544	696	26.89	20,644,294	3,327,869	62.79TB	
Theropithecus	*Theropithecus gelada*	1	Gelada	9565	18	9.15	8,220,974	1,952,648	477.61GB	
Homo	*Homo sapiens*	1	Human	9606	1	1	325,086	37,266	3.26GB	
Gorilla	*Gorilla gorilla gorilla*	1	Western lowland Gorilla	9595	3	16.2	12,878,097	1,976,204	174.93GB	
Mus	*Mus musculus*	14	House mouse	10090	77	30.98	9,347,812	2,568,908	7.59TB	250
Acomys	*Acomys cahirinus*	1	Egyptian spiny Mouse	10068	3	4.67	2,118,569	621,488	231.34GB	

Camarhynchus	*Camarhynchus heliobates*	1	Mangrove finch	1553461	4	6.10	1,087,510	130,547	26.84GB
	Camarhynchus pallidus	1	Woodpecker finch	48878	10	4.79	1,494,843	191,267	52.67GB
	Camarhynchus parvulus	1	Small tree finch	87175	4	5.73	1,463,181	168,340	25.22GB
	Camarhynchus pauper	1	Medium tree finch	93066	4	5.93	1,373,139	160,221	26.09GB
	Camarhynchus psittacula	1	Large tree finch	87178	4	6.15	1,091,827	130,981	27.05GB
Certhidea	*Certhidea fusca*	1	Grey warbler Finch	240232	35	5.79	1,946,612	260,078	222.93GB
	Certhidea olivacea	1	Green warbler Finch	48880	14	5.17	1,568,399	211,363	79.68GB
Geospiza	*Geospiza conirostris*	1	Española cactus Finch	48882	47	0.63	3,347,975	385,013	32.80GB
	Geospiza difficilis	1	Sharp-beaked ground finch	87173	58	6.67	3,565,585	420,037	425.25GB
	Geospiza fortis	1	Medium ground finch	48883	4	5.82	2,136,308	235,124	25.60GB
	Geospiza fuliginosa	1	Small ground finch	48884	8	5.86	3,467,415	382,166	51.53GB
	Geospiza magnirostris	1	Large ground finch	48885	9	5.37	2,657,316	300,196	53.17GB
	Geospiza scandens	1	Common cactus finch	48886	4	5.73	1,857,137	209,928	25.22GB
Loxigilla	*Loxigilla noctis*	1	Lesser antillean bullfinch	1118865	10	6.14	2,628,173	346,215	67.53GB
Melanospiza	*Melanospiza bicolor*	1	Black-faced grassquit	111991	7	5.22	4,693,106	567,755	40.18GB
Pinaroloxias	*Pinaroloxias inornata*	1	Cocos finch	93070	20	4.87	1,157,547	167,868	107.05GB
Platyspiza	*Platyspiza crassirostris*	1	Vegetarian finch	48888	11	5.37	1,491,801	186,322	64.94GB

Hydropotes	*Hydropotes inermis*	1	Chinese water deer	9883	1	113.14	5,542,083	1,218,809	271.53GB	
Muntiacus	*Muntiacus muntjak*	1	muntjak	9888	1	123.89	1,039,587	52,874	297.34GB	
	Muntiacus reevesi	1	Reeves' muntjac	9886	1	132.12	4,321,188	530,570	317.09GB	
	Muntiacus crinifrons	1	Black muntjac	71854	1	203.80	6,028,687	758,535	489.13GB	
Cervus	*Cervus albirostris*	1	White-lipped deer	1088058	1	302.72	7,793,416	1,087,761	726.53GB	
Rangifer	*Rangifer tarandus*	1	reindeer	9870	2	38.98	11,224,394	2,230,502	187.11GB	
Elaphurus	*Elaphurus davidianus*	1	Pere David's deer	43332	2	87.08	3,626,931	823,419	417.99GB	
Giraffa	*Giraffa camelopardalis*	1	giraffe	9894	1	114.95	1,665,964	345,117	275.89GB	
Phoenicopterus	*Phoenicopterus ruber*	1	American flamingo	9217	2	8.5	4,747,729	418,953	20.41GB	
Balaenoptera	*Balaenoptera musculus*	1	Blue whale	9771	1	40.72	8,064,650	1,598,807	105.26GB	
**ALL**	89 species	1086 populations			17360				688.57TB	174945

Note: [1] All 89 species include 16 domestic animals and 73 wild animals; [2] all pig WGS samples include 4337 (1×), 1985 (10×), 83 (20×) and 470 (30×).
